# Investigation of Nonlinear Optical Processes in Mercury Sulfide Quantum Dots

**DOI:** 10.3390/nano12081264

**Published:** 2022-04-08

**Authors:** Vyacheslav V. Kim, Ivan A. Shuklov, Alaa A. Mardini, Arturs Bundulis, Andrey I. Zvyagin, Rawda Kholany, Anna A. Lizunova, Jurgis Grube, Anatolijs Sarakovskis, Oleg V. Ovchinnikov, Rashid A. Ganeev

**Affiliations:** 1 Laboratory of Nonlinear Optics, University of Latvia, Jelgavas 3, LV-1004 Riga, Latvia; vyacheslav.kim@lu.lv; 2 Moscow Institute of Physics and Technology, National Research University, 141701 Dolgoprudny, Russia; aladdin.mardini@phystech.edu (A.A.M.); rawda.kholany@phystech.edu (R.K.); anna.lizunova@gmail.com (A.A.L.); 3 Institute of Solid State Physics, University of Latvia, Kengaraga 8, LV-1063 Riga, Latvia; arturs.bundulis@cfi.lu.lv (A.B.); jurgis.grube@cfi.lu.lv (J.G.); anatolijs.sarakovskis@cfi.lu.lv (A.S.); 4 Department of Physics, Voronezh State University, 394006 Voronezh, Russia; andzv92@yandex.ru (A.I.Z.); ovchinnikov_o_v@rambler.ru (O.V.O.); 5 Tashkent Institute of Irrigation and Agricultural Mechanization Engineers, National Research University, Kori Niyozov Street 39, Tashkent 100000, Uzbekistan

**Keywords:** quantum dots, mercury sulfide, third-harmonic generation, nonlinear refraction, nonlinear absorption

## Abstract

The authors report the third-harmonic generation, nonlinear refraction, and nonlinear absorption in HgS quantum dot (QD) suspensions and films using the nanosecond and femtosecond pulses. High conversion efficiency (7 × 10^−4^) towards the third harmonic (TH) of the 900–1700 nm, 150 fs laser in the thin (70 nm) films containing HgS QDs deposited on the glass substrates is obtained. The authors analyze spectral dependencies of the TH, nonlinear refractive indices, and nonlinear absorption coefficients of QDs in the 500–1700 nm range and discuss the relation between the TH process and the low-order nonlinear optical properties of these quantum dots.

## 1. Introduction

Numerous studies were reported about the routes to synthesize mercury sulfide (HgS) nanoparticles (NPs) and quantum dots (QDs) [1,2,3,4,5,6,7,8,9,10,11,12,13]. At the same time, the nonlinear optical (NLO) characterization of HgS QDs and NPs has rarely been reported [14,15,16,17]. The interest in these properties is related to the applications of HgS QDs and NPs as optical limiters, mode-lockers, and Q-switchers.

In [14], the third-order nonlinear susceptibilities associated with intersubband transition were theoretically calculated for the ZnS/CdSe cylindrical quantum dot quantum well. The nonlinear optical properties of the CdSe/ZnS quantum dot quantum well have been reported in [15]. The third-order nonlinear optical susceptibility induced by the transition between E-1 (inside the well) and E-2 (outside the well) has been calculated for the third harmonic generation (THG) under the effective mass approximation and modified by the local field theory. THG has also been investigated in the ZnS/CdSe/ZnS nanoshell structures [16]. Numerical calculations showed that THG susceptibility has two peaks and depends on parameters such as the size and the kind of structure, the relaxation time, and the pump photon energy. Trigonal HgS, as a highly promising IR NLO material, was analyzed in [17]. Its polycrystalline powder sample exhibits a phase-matched second-harmonic generation response.

In spite of the above theoretical studies, there is a lack of experimental work describing the nonlinear optical properties of HgS QDs, such as Kerr-related nonlinear refraction, nonlinear absorption, as well as generation of third harmonic (TH). Here we analyze the nonlinear optical properties of HgS QD suspensions and films. We describe HgS QDs synthesis, their characterization, properties of colloidal suspensions, and deposition on the thin glass substrates. We report the THG, nonlinear refraction, and nonlinear absorption in HgS QD suspensions and films using the femtosecond and nanosecond pulses. We analyze the dependencies of the THG efficiency, nonlinear refractive indices, and nonlinear absorption coefficients of QDs in a broad spectral range. Finally, we discuss the relation between the THG and the low-order nonlinear optical properties of these QDs.

## 2. Methods and Materials

### 2.1. Experimental Arrangements

**Figure 1:**
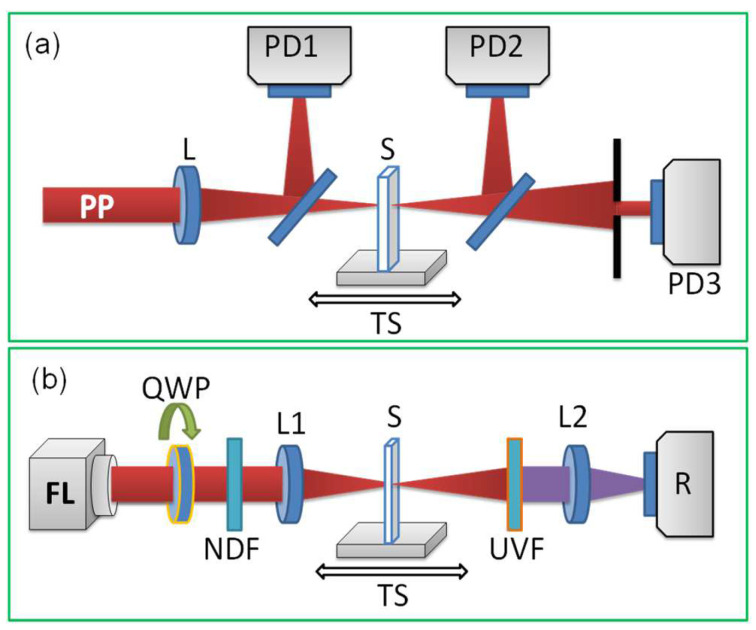
Experimental arrangements for the Z-scans and THG. (**a**) Z-scan scheme. PP, probe pulse (150 fs, 500–1700 nm); L, focusing lens; PD1–PD3, photodiodes (see text); S, sample (70-nm thick HgS QD film on the 0.15-mm thick glass slide); TS, translating stage. (**b**) Experimental setup for third-harmonic generation in thin films. FL: femtosecond laser (150 fs, 100 nJ, *λ* = 900–1700 nm, 500 kHz); QWP: quarter-wave plate; NDF: neutral density filters; L1: focusing lens; S: sample (thin film deposited on the 0.15 mm thick silica glass plate); TS: translating stage; UVF: ultraviolet filter; L2: collecting lens; R: registrar of third harmonic emission (spectrometer USB-2000, Ocean Optics, Ostfildern, Germany).

Two lasers were used to determine the nonlinear optical properties of HgS QD suspensions and films in the femtosecond and nanosecond timescales. The tunable laser source (ORPHEUS-HP + PHAROS PH2 laser, Light Conversion, Vilnius, Lithuania) provided femtosecond probe pulses (PP) during these studies. The 150 fs pulses at 500 kHz repetition rate allowed tuning along the 500–1700 nm spectral region. The standard Z-scan technique was used for the studies of the nonlinear optical properties of HgS QD films and suspensions (Figure 1a). Laser radiation was focused with a 110 mm focal length spherical lens. The sample (70-nm thick film of HgS QDs deposited on the 0.15-mm thick silica glass plate) was moved along the *z*-axis through the focal plane of the spherical lens. The propagated radiation was measured by photodiodes PD2 [open-aperture (OA) scheme] and PD3 [closed-aperture (CA) scheme]. PD1 was used to allow the determination of the normalized transmittances in the case of OA and CA Z-scan schemes. The beam waist diameter (FWHM) of the laser beam was 28 μm in the case of the focusing by a 110 focal length lens. The intensity of this beam did not exceed 5 × 10^10^ W cm^−2^, while the optical damage of the HgS QD film by femtosecond pulses occurred at ~1 × 10^11^ W cm^−2^.

These studies allowed the authors to determine the spectral dependencies of the nonlinear refractive index (*γ*) and nonlinear absorption coefficient (*β*) of HgS QDs being presented in the solid-state (thin films) phase. The CA Z-scan scheme was calibrated using the known value of the nonlinear refractive index of carbon disulfide.

The analysis of nonlinear refraction, saturable absorption (SA), reverse saturable absorption (RSA), and two-photon absorption (2PA) using long (nanosecond) pulses can define the spectral peculiarities of those processes in QDs, which could not be revealed using the short laser pulses. To determine the NLO response of our samples in the case of long PP, the authors used the 10 ns pulses from the Nd: YAG laser at the two wavelengths (1064 and 532 nm). A similar Z-scan scheme was used.

Laser radiation was focused using a 300 mm focal length lens. We used the beam profiler with a CinCam CMOS Nano 1.001 camera (Axiom Optics, Los Angeles, CA, USA) to characterize the beam waist of the focused laser radiation. A beam profiler sensor was placed in the focal spot position. A set of ND filters was used to prevent the damage of the sensor. The position of the beam profiler was scanned along the direction of beam propagation and the minimum beam waist was recorded. The beam waist diameters were 80 μm and 60 μm (at half width of 1/e^2^ maximum of the spatial distribution at the focal plane) in the case of fundamental (1064 nm) and second-harmonic (532 nm) beams, respectively. The 0.2-mm-thick fused silica cell containing HgS QD colloidal suspension, or HgS QD thin film (70 nm) deposited in the glass substrate, was moved along the *z*-axis through the focal point using a translating stage controlled by a computer. The intensities of 1064 nm and 532 nm pulses used in the experiments did not exceed 9 × 10^8^ W cm^−2^ and 7 × 10^8^ W cm^−2^, respectively, to avoid the breakdown of the suspension and film.

Special attention was paid to the CA Z-scans. To collect these scans, the 1-mm aperture allowing propagation of ~5% of radiation was fixed at a distance of 150 cm from the focal plane, behind which the photodiode was located. The scheme with CA allowed for the determination of the sign and value of the nonlinear refractive index of the QD-containing medium. The OA scheme allowed for the determination of the nonlinear absorption of the sample.

The optical damage thresholds of suspensions and films using 10 ns, 532 nm and 10 ns, 1064 nm PP were determined to be 6 × 10^9^ and 9 × 10^9^ W cm^−2^, respectively. These values of optical damage were more than one order of magnitude larger than the intensities used during our measurements of the optical nonlinearities of samples.

The standard fitting procedure was applied to the measured Z-scans, when commonly used relations [18] for determination of the *γ* and nonlinear absorption coefficients attributed to the 2PA (*β*_2PA_), SA (*β*_sat_), and RSA (*β*_RSA_), as well as saturated intensity (*I*_sat_), were applied to fit our experimental data.

The scheme for the measurements of TH yield was almost similar to the Z-scan scheme (Figure 1b). For these experiments, we used the thin film containing HgS QDs, similar to the one used during measurements of the nonlinear refraction and nonlinear absorption. The used parameters of the ORPHEUS-HP + PHAROS PH2 femtosecond laser (Light Conversion) were as follows: 150 fs pulse duration, 50 nJ pulse energy, 500 kHz pulse repetition rate. The wavelength of output radiation from the laser allowed for tuning in the range of 190–2500 nm, while THG was analyzed in the 900–1700 nm range of laser radiation. More details of THG experiments are described in Section 5.

### 2.2. Preparation and Characterization of Samples

Mercury sulfide was synthesized by applying a solution of sulfur in oleylamine using the modified procedure described in [19]. The synthesized colloidal QDs with the first absorption peak at 1036 nm were applied for the preparation of thin films. In the typical synthesis, two solutions were prepared: solution A: 30 mg (0.11 mmol) of HgCl_2_ and 6 mL of dry oleylamine were placed in a round bottom flask with two necks under argon flow at room temperature and gradually heated to 100 °C for 1 h, and solution B: sulfur precursor stock solution was prepared by dissolving 1 mmol (0.032 g) of S in 10 mL of oleylamine under Ar flow at 100 °C for 1 h. Then the solutions were cooled to 60 °C and 2 mL of sulfur precursor was rapidly injected by syringe into the solution A under argon flow. The reaction mixture was stirred at 60 °C for 15 min. After that, a quenching mixture consisted of 16 mL tetrachloroethylene (TCE), 3.1 mL (0.8 mmol) dodecanthiol, and 1.5 mL tri-n-octylphosphine was added to the reaction mixture and simultaneously cooled in the ice bath.

4 mL of methanol was added per 6 mL of the resulting solution and centrifuged to isolate the precipitate. The resulting precipitate was redispersed in 2 mL of TCE, and 2 mL of the acetonitrile was added to the resulting solution, which was again centrifuged. The precipitate was dried under Ar flow, redispersed in 0.5 mL of TCE, and filtered using 0.22 µm hydrophobic filter. 0.2 mL of this solution was diluted to 2 mL by adding TCE and characterized by using the UV-visible/NIR spectrophotometer (JASCO V-770, Los Angeles, CA, USA).

The thin films were prepared by layer-by-layer deposition using the dip-coating on a 0.15 mm-thick glass substrate. The ligand shell exchange by 1,2-ethanedithiol (EDT) has been done using 1:1:100 EDT/HCl/2-propanol solution [20]. The glass substrate was withdrawn at a speed of 18 mm/min. Excess of exchanging reagent was removed by washing the thin films in 2-propanol. The procedure was repeated 5 to 20 times in order to obtain films with thicknesses of ~70 nm. The position of the first absorption peak maximum was shifted toward the longer wavelength range in the film after ligand exchange. In the case of EDT, it was 16 nm red shifted towards 1051 nm in the case of the film.

**Figure 2:**
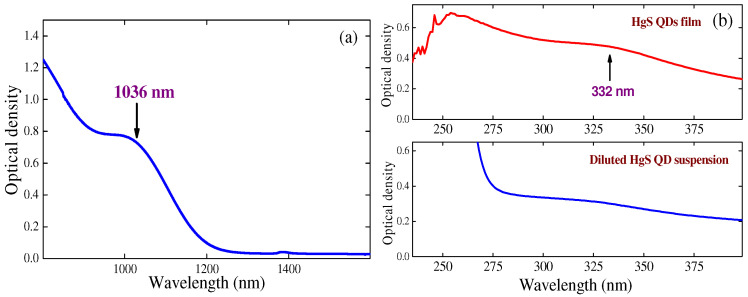
Absorption spectra of HgS QD film and suspension. (**a**) Optical density of the suspension contained in 1 mm thick silica glass cell in the near infrared range. (**b**) Upper panel: UV absorption spectrum of the 70 nm thick film contained HgS QDs. Bottom panel: UV absorption spectrum of the diluted suspension.

The optical density of the HgS QD suspension contained in 1 mm-thick silica glass cell is shown in Figure 2a for the near IR range. One can see the appearance of the absorbance band centered at 1036 nm. The UV absorption spectrum of the 70 nm thick film contained HgS QDs is presented in the upper panel of Figure 2b. The characteristic band centered at 332 nm is seen. The diluted suspension steadily increased the optical density down to 275 nm and then showed a significant growth of optical density at shorter wavelengths (bottom panel of Figure 2b).

**Figure 3:**
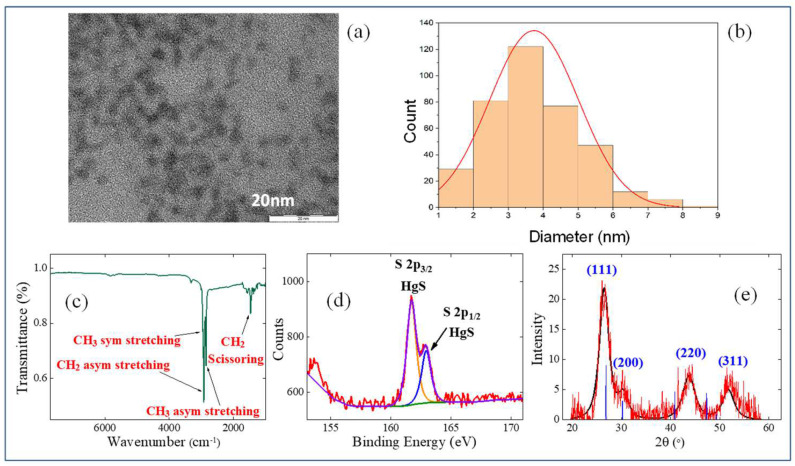
Characterization of sample. (**a**) TEM of HgS QDs. (**b**) Size distribution histogram. (**c**) FTIR spectrum. (**d**) High-resolution XPS. (**e**) XRD of sample.

The shape and size of the resulting HgS QDs were determined using the transmission electron microscopy (TEM, JEM-2100, JEOL GmbH, Freising, Germany). It was shown that the mean size of the resulting HgS QDs is 4 nm (Figure 3a,b), which matches well with the first absorption peak in the range of ~1037 nm in the TCE solution, according to earlier testified data of the spherical HgS QDs [21].

Fourier-transform infrared spectroscopy (FTIR, Spectrum 100, PerkinElmer, Waltham, MA, USA) in the 8000–1000 cm^−1^ range was used for analyzing the ligand shell (Figure 3c). The presence of bounded 1-dodecanethiol was confirmed by FTIR. C–H stretching of methyl and methylene groups was detected at 2956 cm^−1^, 2922 cm^−1^, and 2852 cm^−1^, and the bending of methylene groups was demonstrated at 1466 cm^−1^. Non-bounded thiol was not observed in the FTIR spectrum.

The composition of HgS QDs was carefully examined using the X-ray photoelectron spectroscopy (XPS) and X-ray diffraction (XRD). XPS measurements (PHOIBOS 150 MCD, SPECS Surface Nano Analysis GmbH, Berlin, Germany) were employed using the Mg Ka radiation (1253.6 eV). The high-resolution XPS spectrum (Figure 3d) proved that mercury sulfide is the essential component of the monocrystalline core. The XPS spectrum with typical binding energy values of 161.7 eV (S 2p_3/2_) and 162.8 eV (S 2p_1/2_) was compatible with the S^2−^ spectra.

Since the synthesized species could suffer from oxidation in air, the possible oxidation was carefully analyzed. The sulfur is only one chemical element that could be oxidized in HgS, while the mercury is already in the highest oxidation state (Hg^2+^). Therefore, the high resolution XPS spectrum in 155–165 eV region is the most representative to track the possible oxidation products. No higher sulfur oxidation states were detected by XPS. XRD (ARL X’TRA X-ray powder diffractometer, Thermo Fisher Scientific Inc., Waltham, MA, USA) using CuKa radiation was applied to identify the crystalline structure of the obtained HgS nanocrystals. The XRD spectrum (Figure 3e) of HgS QDs showed a single crystalline phase of cubic *β*-HgS (metacinnabar) with the peaks corresponding to the crystal faces (111), (200), (220), and (311). The broadened signals are typical for the small-sized NPs (d < 5 nm), which indicate the diminished sizes of the particles. The average crystallite diameter was estimated to be 3.7 nm from the XRD pattern using the Debye–Scherrer formula, which matches well with the TEM measurements (Figure 3b). Other crystalline phases of HgS were not present. Mercury oxide (HgO) (orthorhombic), which could be formed by heavy oxidation of HgS samples, was absent in the obtained XRD spectrum. Thus, based on the XPS and XRD data, as well as FTIR measurements, one can exclude a significant oxidation of the synthesized samples. It could be concluded that the semiconductor core of QDs consists of non-oxidized *β*-HgS.

## 3. Measurements of the Nonlinear Optical Refraction and Nonlinear Absorption of HgS QD Samples

### 3.1. Application of Femtosecond Probe Pulses

**Figure 4:**
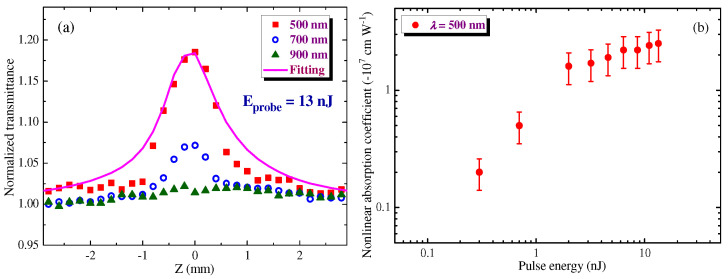
Results of the OA Z-scan studies of HgS QD film. (**a**) OA Z-scans using 500 (red filled squares), 700 (blue empty circles), and 900 nm (green filled triangles) at similar a energy of the probe pulses (13 nJ). Solid curve corresponds to the fitting of the OA Z-scan in the case of 500 nm PP. (**b**) The dependence of the nonlinear absorption coefficient responsible for saturable absorption at *λ* = 500 nm on the energy of the probe pulses.

SA was a dominating nonlinear absorption process observed during the whole set of the OA Z-scan studies of HgS QD film using femtosecond probe pulses (Figure 4a). We did not observe the positive nonlinear absorption in this sample during experiments using femtosecond pulses. Neither 2PA nor RSA played a significant role in the case of application of the 150 fs PP varying by energy (intensity) between 0.3 and 15 nJ (1 × 10^8^–5 × 10^9^ W cm^−2^). The analysis of the OA Z-scans along the whole range of spectral variations of the PP allowed for conclusions to be drawn about the spectral dependence of the wavelength of laser pulses on the *β*_sat_. The above-mentioned used intensities during these studies were far less than the breakdown threshold of this film (~1 × 10^11^ W cm^−2^), thus pointing out the involvement of SA in the bleaching of the studied samples. The application of notably stronger pulses (120 nJ) led to the appearance of visible damage on the surface of the film after a whole set of measurements.

In accordance with the model of SA, the relaxation rate of excitations should not depend on the laser intensity. The absorption rate A is determined by the parameter A = *α*_0_/(1 + *I*/*I*_sat_). Here, *I* is the variable intensity along the *z*-axis during focusing of laser radiation and *α*_0_ is the linear absorption coefficient. The *I*_sat_ and *α*_0_ depended on the concentration of the active centers in the medium, the effective cross-sections, and the lifetime of the excitations.

Figure 4a shows a set of OA Z-scans of the 70 nm-thick film deposited on the 0.15 mm-thick silica glass plate. The authors present the results of studies only for the 500, 700, and 900 nm PP since the longer-wavelength radiation did not show the nonlinear absorption. All scans were carried out at the same energy of 150 fs pulses (13 nJ, *I*_0_ ≈ 4 × 10^9^ W cm^−2^). The spectral tuning of PP was performed with a step of 200 nm. One can see that the bleaching of the film steadily decreased with the growth of the wavelength from 500 to 900 nm.

The fitting of OA Z-scans was used for defining the nonlinear absorption coefficient related to the SA (*β*_sat_). Figure 4b shows the dependence of the *β*_sat_ on the energy of the 500 nm PP. As was expected, this parameter weakly depended on the pulse energy in the case of 2–15 nJ PP. At smaller energies, SA was less pronounced.

The main contributions to the uncertainty in determination of the nonlinear absorption coefficients and nonlinear refractive indices arose from the power, the pulse duration, and the beam waist radius measurements, laser power fluctuations, and uncertainty in the fitting procedure. One can also assume the variations of these parameters for different used wavelengths of the probe radiation. Including all error sources, the uncertainty in the measured values was estimated to be 30%, which is typical for experimental errors during Z-scan measurements.

The fitting of OA Z-scans determined the *β*_sat_ of the film in the case of 150 fs PP [*β*_sat_ = −(2 ± 0.6) × 10^−7^ cm W^−1^) in the case of 500 nm PP]. This parameter was varied between −0.8 × 10^−7^ cm W^−1^ (at *λ* = 700 nm) and −2 × 10^−7^ cm W^−1^ (at *λ* = 500 nm), so the bleaching significantly depended on the wavelength of PP. The corresponding saturation intensity was determined to be *I*_sat_ ≈ 8 × 10^10^ W cm^−2^ in the case of 500 nm PP. One can see that the thin film containing HgS QDs demonstrates a relatively low value of saturated intensity. The low-*I*_sat_ saturable absorbers may allow the formation of the low-power femtosecond laser sources. The saturation intensity of light at which the extinction coefficient is reduced by a factor of 2 is an important parameter of such lasers. SA does not directly follow the spectral absorption dependence since the former process depends on different factors related to the structure of energy levels of the studied material. Meanwhile, one can admit that *β*_sat_ increased with the steady growth of the linear absorption in the visible range.

**Figure 5:**
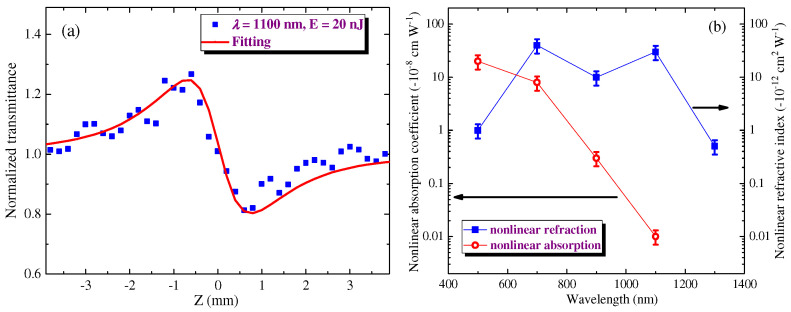
Analysis of the nonlinear refractive properties of the film. (**a**) CA Z-scan using 1100 nm, 20 nJ, and 150 fs probe pulses. Solid curve: fitting of CA Z-scan. (**b**) Spectral dependencies of the nonlinear refractive index (blue-filled squares) and SA-related nonlinear absorption coefficient (red, empty circles).

Figure 5a shows the CA Z-scan of film using 1100 nm, 20 nJ, and 150 fs pulses at 500 kHz repetition rate. The fitting of the CA Z-scan (Figure 5a, red solid curve) helped determine the nonlinear refractive index of the studied film. The discrepancy between the fittings and experimental CA and OA curves shown in this and other figures can be attributed to some asymmetric (slightly elliptical) shape of the used beam in the focal area (see the discrepancy in the case of the negative and positive values of z). The film demonstrated self-defocusing along the whole range of studied pulse energies variations (0.5–30 nJ). The negative nonlinear refraction was observed in a broad spectral range (500–1300 nm; Figure 5b, blue filled squares). The largest values of the negative nonlinear refractive index were observed in the 700–1100 nm range [*γ* = −3 × 10^−11^ cm^2^ W^−1^], with a further drop in this parameter by more than one order of magnitude at 500 and 1300 nm. Notice that another NLO parameter, the negative nonlinear absorption coefficient *β*_sat_, steadily decreased towards the longer-wavelength region (Figure 5b, red empty circles) leading to an almost complete disappearance of SA at *λ* = 1100 nm.

In all of the CA experiments with the studied film, the authors observed negative nonlinear refraction. One can assume that the application of 150 fs PP at a high pulse repetition rate (500 kHz) can cause a thermal lens formation. Meanwhile, the thin film did not form a thermal lens due to the accumulative effect. To prove this assumption, we analyzed the CA Z-scan of film using the quarter-wave plate (QWP) placed in front of the focusing lens to determine the role of the heating processes in the formation of the thermal lens leading to the self-defocusing in thin film. The application of circularly polarized 1100 nm pulses did not reveal the similar pattern of the Z-scan obtained in the case of the linearly polarized beam (Figure 5a). Correspondingly, the thermal lens, which does not depend on the polarization of the heating radiation, was not formed during these experiments using 150 fs, 20 nJ, 500 kHz, and 1100 nm laser pulses. As a consequence of these studies, the Kerr effect can be assumed as the alone process responsible for the observed self-defocusing of 1100 nm, 150 fs pulses in the HgS QD film. The authors’ separate analysis of the NLO of a pure 0.15 mm-thick silica glass plate did not show any NLO effect in all the ranges of intensity and wavelength variations used in the present studies.

### 3.2. Application of Nanosecond Probe Pulses

The use of HgS QD suspension placed in the silica glass cell led to the growing influence of the self-modulation of the propagated radiation in the case of femtosecond PP. Meanwhile, this process was significantly diminished in the case of nanosecond PP. Below, the authors report the NLO studies of the suspensions and films containing HgS QDs.

**Figure 6:**
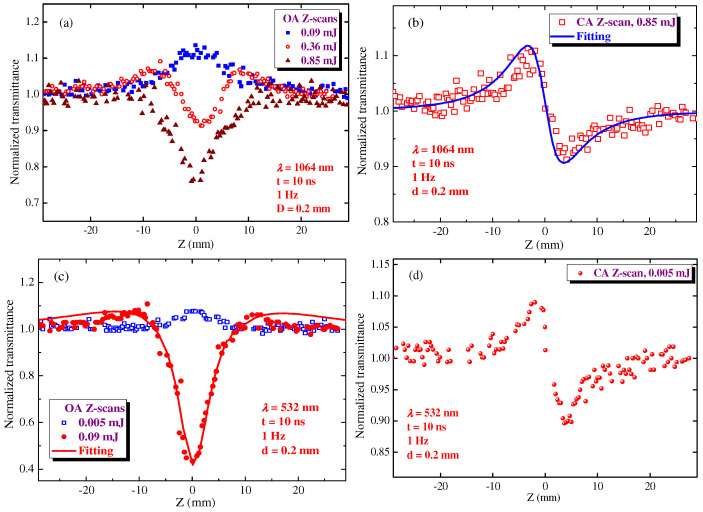
OA (**a**,**c**) and CA (**b**,**d**) Z-scans of 0.2 mm-thick HgS QD suspension using 10 ns probe pulses. (**a**,**b**) 1064 nm probe pulses. (**c**,**d**) 532 nm probe pulses. The energies of probe pulses are shown on the graphs. Solid curves correspond to the fittings of CA and OA Z-scans.

The colloidal HgS QD suspension was placed in the 0.2-mm thick silica glass cell and analyzed by the Z-scan technique. Contrary to the application of femtosecond PP for the studied thin (70 nm) films, the longer pulses allowed for the observation of the dynamics of the nonlinear absorption in the extended (0.2 mm) medium, when initially, the manifested SA was replaced by the RSA once the energy of 1064 nm, 10 ns PP exceeded some threshold (Figure 6a). An even stronger modification of the OA Z-scans was observed in the case of the shorter-wavelength PP (532 nm, Figure 6c). The threshold of this modification was notably different for the used laser beams. The use of 0.09 mJ, 1064 nm pulses led to the observation of the only process (SA; Figure 6a, blue filled squares), while the same energy of 532 nm pulses caused the almost entire suppression of SA and the dominance of the RSA (Figure 6c, red filled circles). The fittings of the latter scans allowed determination of the nonlinear absorption coefficients attributed to the SA and RSA (*β*_sat_ = −(6 ± 1.8) × 10^−8^ cm W^−1^ and *β*_RSA_ = (5 ± 1.5) × 10^−7^ cm W^−1^) in the case of 532 nm, 10 ns PP.

Among different models determining the SA, which is a dominant mechanism in the case of the weak probe pulses, are those with a the two-level system possessing heterogeneously broadened states, a kinetic model, and an empirical model [22,23]. In the case of the kinetic model, the absorption coefficient can be presented as *α*(z) = *α*_0_/(1 + *I*(z)/*I*_sat_), where *I*(z) is the variable intensity of laser pulses. This model showed the best fit with our experimental data. The relations of this model determined the saturated intensity (*I*_sat_ = 7 × 10^8^ W cm^−2^ in the case of 10 ns, 1064 nm PP and *I*_sat_ = 2 × 10^8^ W cm^−2^ in the case of 10 ns, 532 nm PP).

The corresponding negative nonlinear absorption coefficient in the case of 1064 nm pulses was calculated to be *β*_sat_ = −4 × 10^−9^ cm W^−1^. This parameter did not change with the growth of laser intensity in the range of 0.02 and 0.16 mJ energies of the 1064 nm PP. The application of 0.2 mJ pulses led to the uncertainties in the determination of *β*_sat_ due to the growing influence of the RSA dominating at larger energies of PP in the OA Z-scans (Figure 6a). 2PA or RSA are the commonly reported mechanisms of the nonlinear absorption of QDs. Notice that, in most cases, SA follows by RSA. HgS QD suspension did not show any absorption bands in the region of the twice shorter wavelength than the wavelength of the PP (1064 nm). Considering the fact that we did not see the excitonic band attributed to QDs at the lower wavelengths of PP, one can assume that 2PA of 1064 nm pulses hardly can be considered as an alternative to the RSA mechanism for the explanation of the growth of positive nonlinear absorption observed in our studies. The fitting of the OA Z-scan presented in Figure 6a (brown filled triangles) in the case when the RSA entirely dominates over the SA determined *β*_RSA_ at *λ* = 1064 nm to be 1 × 10^−8^ cm W^−1^.

The possibility of the dynamic scattering of the nanosecond PP by QDs films has recently been studied using the 10 ns pulses [24]. Those studies did not observe the nonlinear scattering in the case of 532 nm probe pulses. The authors analyzed the possibility of dynamic scattering of the probe pulses in colloidal HgS QDs by installing the additional photodiode out of the optical axis of beam propagation. These measurements showed the pattern of very weak and unchanged scattering during the propagation of our sample (0.2 mm-thick cell filled in with QD suspension) through the focal plane of the focusing lens while probing the whole range of used intensities of laser pulses. No valley was observed during our OA Z-scans as well, which point out the insignificance of the scattering process in the studied suspensions. The same can be said about films. Thus, the nonlinear scattering cannot be considered as the mechanism of the decrease of normalized transmittance at the larger intensities of 532 nm probe pulses. The same conclusion was obtained in the case of a twice-as-long wavelength of the probe pulses. This conclusion may also be applied to the measurements in the case of femtosecond PP.

CA Z-scan studies were also performed using different energies of the probe pulses. At *λ* = 1064 nm, the nonlinear refraction was observed only in the case of relatively strong laser pulses. Figure 6b shows the CA Z-scan in the case of 0.85 mJ PP, though the self-defocusing was observed at smaller energies of laser pulses. The fitting of this curve determined *γ* at *λ* = 1064 nm (−1 × 10^−13^ cm^2^ W^−1^). Similar fitting of the CA Z-scan using 532 nm showed that the self-defocusing at this wavelength is significantly stronger (Figure 6d, −6 × 10^−13^ cm^2^ W^−1^) compared with the case of the 1064 nm pulses. One can see that the approximately similar peak-to-valley values shown in these two CA curves (Figure 6b,d) were obtained using the 0.85 and 0.005 mJ pulses.

To confirm that the NLO properties are purely related with the HgS QDs, the main solvent (TCE) was analyzed at two wavelengths (532 and 1064 nm) of probe radiation using a similar procedure of Z-scan. No NLO response was observed during this test study, which indicated that the observed processes were derived entirely from the HgS QDs. Notice the absence of the thermal lens building in the pure TCE representing the main liquid component of the studied suspension. Similarly, the self-defocusing observed in these studies of the colloidal suspension of the HgS QDs cannot be attributed to the thermal lens building due to the low pulse repetition rate (10 Hz). The same conclusion was made during similar studies of pure TCE using femtosecond pulses.

In the case of relatively long pulses, the molecular Kerr-related nonlinearities can prevail over electronic Kerr-related processes. Commonly, the former refractive nonlinearities demonstrate the self-defocusing properties. The prevalence of the molecular nonlinearities related to the reoriented mechanisms over the electronic nonlinearity may play an important role in the picosecond, and especially, in the nanosecond timescale. Thus, the larger values of the *γ* of studied samples in the case of nanosecond PP with regard to the 150 fs pulses could be expected. Notice that we probed the films and suspensions possessing approximately similar optical densities. Meanwhile, the self-defocusing in the film probed by femtosecond pulses was stronger than the one in the colloidal suspension probed by nanosecond pulses. This observation may reveal the assumption that the same amount of QDs being combined at a smaller volume (70 nm thin film) can enhance the NLO processes due to the growing influence of the combined local field effect compared with the same amount of QDs distributed along the much wider region (0.2 mm).

**Figure 7:**
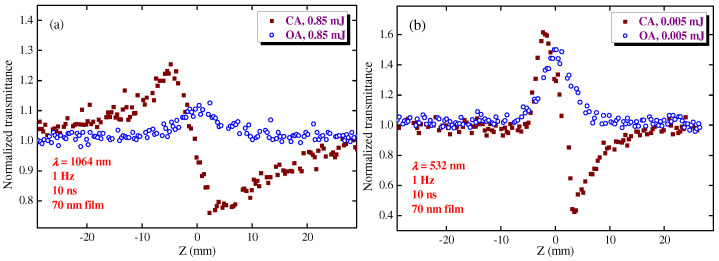
Z-scans of 70 nm-thick HgS QD film using 10 ns probe pulses. (**a**) 1064 nm probe pulses. (**b**) 532 nm probe pulses. The energies of probe pulses are shown on the graphs.

To prove this assumption, the authors performed similar studies of films using the 10 ns pulses. Figure 7 shows the corresponding Z-scans of HgS QD film using the 1064 (Figure 7a) and 532 nm (Figure 7b) pulses. A comparison of the OA Z-scans in the case of the longer-wavelength femtosecond pulses (Figure 4a) and nanosecond pulses (Figure 7a) showed the stronger NLO response in the latter case. Actually, there was a very weak SA in the case of 1000 nm, 150 fs pulses. As for the visible (532 nm) region, approximately the same SA was observed in the case of 150 fs and 10 ns pulses, in spite of a large difference in the applied intensities of PP. Meanwhile, no RSA was registered in the film at both wavelengths (Figure 7) contrary to the suspension (Figure 6a,c). The NLO optical parameters of film were determined to be the *β*_sat_ = −4 × 10^−8^ and *β*_sat_ = −9 × 10^−8^ cm W^−1^, as well as *γ* = −3 × 10^−13^ and *γ* = −2 × 10^−12^ cm^2^ W^−1^ at *λ* = 1064 nm and *λ* = 532 nm, respectively. One can see that these values measured in the film exceed those measured in the 0.2 mm-thick cells filled in by the HgS QD colloidal suspension, which proved our assumption regarding the prevailing NLO properties of the thin films with regard to the sparsely distributed QDs of the same density.

## 4. Third-Harmonic Generation in Thin Films

**Figure 8:**
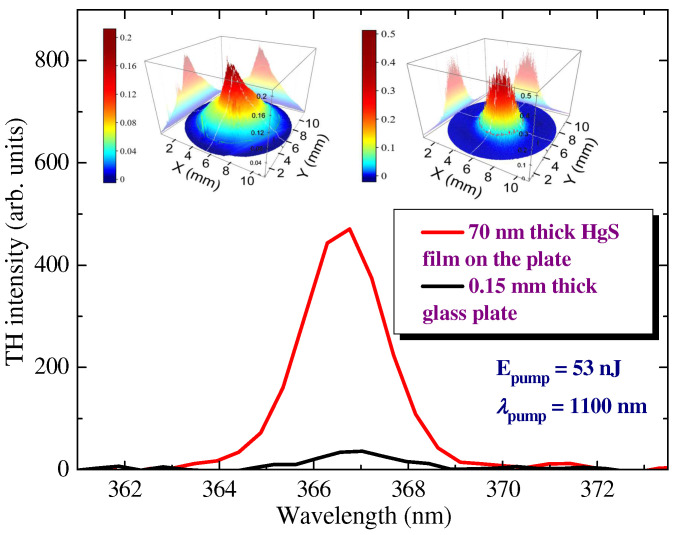
Characteristics of third-harmonic emission. Spectra of the third-harmonic emission from the pure silica glass substrate (d = 0.15 mm, black solid curve) and thin film (d = 70 nm) of HgS QDs deposited on the silica glass substrate (red solid curve). Inset: 3D spatial distributions of the third harmonic (right panel) and fundamental (left panel) beams.

Below are the results of studies when the authors used the fixed wavelength of the fundamental radiation (1100 nm) from the femtosecond laser to measure different characteristics of the THG process (Figure 8). The spatial shapes of the fundamental and TH beams were measured at a distance of a few centimeters from the focal plane (inset to Figure 8). Initially, we measured the TH emission from the pure 0.15 mm-thick silica glass plate (Figure 8, black dotted curve) and then collected the same emission in the case of the film + plate combination (red solid curve).

The intensity of TH from the pure glass plate was more than one order of magnitude smaller than in the case of the plate containing thin (70 nm) film of HgS QDs. Notice that the ratio of the thicknesses of silica glass plates and films was 0.15 mm/70 nm ≈ 2 × 10^3^. Correspondingly, one can expect a ~4 × 10^6^ drop of the NLO response of glass at similar thickness as the HgS QDs film, once the authors assume a quadratic dependence of the TH yield on the thickness of the studied species. Thus, the THG efficiency in the studied film was estimated to be at least 4 × 10^7^ times larger than in the silica glass plate of similar thickness. Both experiments were carried out at the same energy of the pump pulses (53 nJ). The three-fold increase of PP energy (~150 nJ, *I* = 1 × 10^11^ W cm^−2^) led to the optical damage of the film.

The central wavelength of TH in all these experiments with thin film and pure silica glass was ~366.8 nm, which almost corresponded to the exact position of the tripled frequency of the used laser pulses (366.6 nm). The intensity of fundamental radiation in the focal plane was 3 × 10^10^ W cm^−2^. One can assume that, at this intensity, no self-modulation can be expected during the propagation of the fundamental radiation through the thin glass and ultrathin film. In the case of film + glass composition, the notably stronger emission of TH from the ultrathin film was combined with the TH emission from the substrate.

The inset in Figure 8 shows the images of 367 and 1100 nm beams measured at the same position after the defocusing of the converting and converted radiation 100 mm from the focal plane of the focused lens. The divergence of TH was approximately three times smaller compared with the fundamental beam (*λ* = 1100 nm). The shape of the TH beam was close to Gaussian.

**Figure 9:**
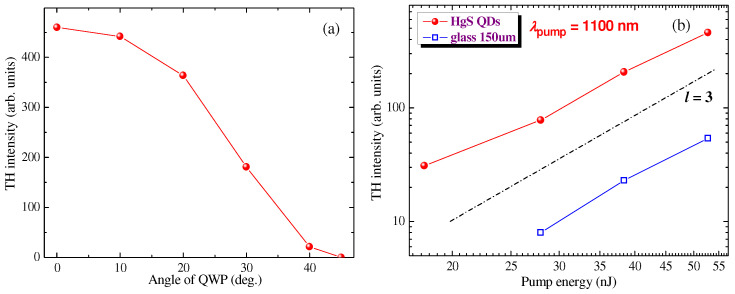
Polarization and intensity dependencies of TH yield from the samples. (**a**) Variation of the TH yield on the rotation angle of the quarter-wave plate (QWP) placed in front of the focusing lens. At the 45° rotation of QWP corresponding to the circular polarization of the fundamental beam, the TH emission was not observed. (**b**) TH intensity as a function of the pump radiation energy. Red diamonds and blue, empty squares show the *I*_TH_ (*I*_PP_) dependencies in the case of film + plate and pure plate, respectively. The black, dot-dashed line corresponds to the expected cubic slope of these dependencies (*l* = 3; *I*_TH_ ∞ *I*_PP_^3^).

The polarization dependencies of TH yield from pure glass and glass + film showed decay with the deviation of the polarization state from linear to elliptical circular when the harmonic emission was entirely disappeared in the latter case (Figure 9a). Here, the authors show the variation of the TH yield from the glass + film sample during the rotation of the QWP placed in front of the focusing lens. At the 45° rotation of QWP corresponding to the circular polarization of the fundamental beam, the TH emission entirely disappeared.

The “Z-scans” of film + plate sample showed a fast decrease in TH yield out of the focal plane of the focused lens. The width of those z-dependent curves was determined by the small Rayleigh length of the used fundamental radiation (0.3 mm). The experiments using the film + plate and pure plate samples followed the cubic dependence of the TH yield on the pulse energy along the whole range of the used pulse energy variations (15–55 nJ, Figure 9b).

**Figure 10:**
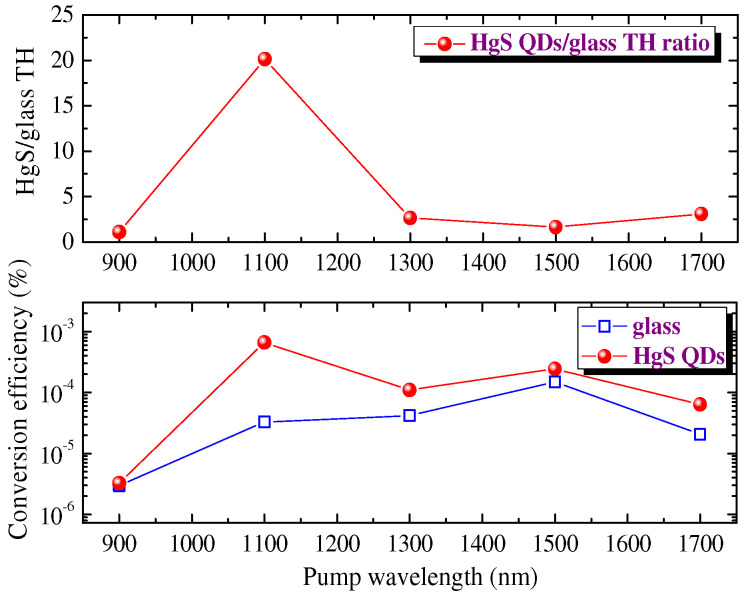
Spectral dependencies of the TH yields from HgS QD film and pure silica glass plate. (**Upper panel**): the ratio of TH yields from the film + plate and pure plate. (**Bottom panel**): absolute values of TH conversion efficiencies in the case of film + plate (red diamonds) and plate (blue, empty squares).

The spectral-dependent measurements of the absolute values of TH conversion efficiencies along the range of tuning the fundamental radiation were performed using the calibrated filters and spectrometer. The spectral measurements of THG in film + plate and pure plate were performed by tuning the wavelength of the femtosecond laser (Figure 10). The laser allowed the generation of radiation in two regions (320–600 nm and 700–2200 nm). The authors restricted the measurements of TH to the 900–1700 nm spectral range of fundamental pulses variations. The reason for restricting the shorter wavelength fundamental pulses was related to the growing absorbance of the generated TH in the region below 300 nm. The limitation in the use of longer-wavelength fundamental pulses (λ > 1700 nm) was caused by the decreased energy of those pulses compared with the shorter-wavelength radiation. The upper panel of Figure 10 shows the ratio of TH yields from the film + plate and pure plate. One can see a significantly larger difference between the TH yields in the region of 1100 nm of the fundamental radiation (TH_HgS+glass_/TH_glass_ = 20) compared with other regions where the efficiency from the former sample was only 2 to 3 times higher compared with the THG in puller silica glass plate. The authors again underline the incomparable difference in the thicknesses between the glass and HgS QD film, which caused relatively small TH_HgS+glass_/TH_glass_ ratios.

THG conversion efficiency in the films at *λ*_pump_ = 1100 nm was measured to be 7 × 10^−4^. The error factor of these measurements of the absolute value of conversion efficiency was estimated to be equal to two. The relatively strong conversion efficiency in such thin films can be attributed to the influence of the quantum confinement effect in the case of small-sized species, such as QDs, when the local field can lead to the stronger NLO response of these tiny species. Such an effect has earlier been reported in the comparative studies of the species of different sizes. Smaller particles demonstrated stronger NLO response compared with the larger species of the same material.

## 5. Discussion

The goal of the present study was to determine the low-order optical nonlinearities of mercury sulfide quantum dots. One can consider a connection between them and higher-order NLO responses of this material. In particular, the high-order harmonics can efficiently generate in the media containing the QDs possessing strong nonlinear refraction and absorption. Are the HgS QDs also suitable for increasing the efficiency of high-order harmonics generation (HHG)? Previously, the application of metal sulfide QDs as the emitters of high-order harmonics of ultrashort laser pulses achieved the efficient conversion of IR emission of Ti: sapphire lasers towards the shorter-wavelength region (*λ* < 50 nm) [25,26].

Obviously, the usefulness of these species is not limited to the application of HHG. The potential applications of the colloidal suspensions and films containing the HgS QDs showing strong SA may include their use as the mode-lockers or Q-switchers in lasers. Another option is the application of their strong RSA at relatively high laser intensities in the optical limiting devices for the protection of the sensitive registrars and eyes.

To the best of our knowledge, no studies were reported on the lowest-order harmonic generation in the QDs-containing films. QDs demonstrate intermediate properties between bulk semiconductors and discrete atoms or molecules. Their characteristics change as a function of both size and shape. For these reasons, novel synthesized semiconductor QDs require examination under different conditions using laser pulses of variable energies, wavelengths, and durations to understand different NLO mechanisms and to reveal their attractive properties for practical applications. In recent years, there has been an increasing interest in the study of the NLO properties of semiconductor QDs with average sizes of less than 4 nm. The main advantages of such NLO media are high optical uniformity of the colloidal solutions and films, as well as size-dependent spectral and luminescent properties related with the confinement effect. The dimensional dependence of the transition energy in absorption and luminescence opens up possibilities for achieving resonances, the use of which can reduce the threshold for the observation of the optical nonlinearities.

In the case of low-order harmonic generation, two perspectives can be considered: on one hand, it can be useful in the search for new efficient materials that will act as nonlinear media in THG and higher-order processes. On other hand, this technique is suitable as a diagnostic tool to characterize the species presented in laser-induced plasmas. Particularly, the shape of the harmonic distribution can reveal the presence of some elements incorporated in the ablating targets. Such an opportunity has been demonstrated using the ablation of the stainless-steel nozzle of gas jet during the studies of HHG in argon gas [27]. Without the ablating nozzle by picosecond pulses, the harmonic spectrum represented the ordinary spectrum of gradually decreasing harmonics from the Ar gas jet. The ablation of the nozzle led to an appearance of the components of stainless-steel ablation in the axis of the driving beam propagation. These components drastically changed the HHG spectrum. The harmonics from H13 to H25 generated in the mixture of gas and plasma were five times stronger than those generated in the pure argon gas. The most intriguing pattern was observed in the area of harmonic cutoff. It was shown that the 27th harmonic almost disappeared compared to the neighboring ones. Meanwhile, the following harmonic, H29, was notably stronger that H25, which is an unusual case.

Stainless steel contains a number of constituents that define its structure, hardness, and other physical and chemical properties. It was shown that one of the important constituents was the chromium (12%). The role of Cr in the harmonic spectrum generated in stainless steel plasma has been studied earlier using pure Cr and pure Fe as the targets for HHG experiments [28]. The former plasma has shown a considerable enhancement of the intensity of the 29th harmonic. Thus, the abovementioned studies have demonstrated that the increase of the intensity of the 29th harmonic, in the case of ablated stainless steel, was associated with the presence of the chromium in the ablating sample.

The search for methods to ensure a stable concentration of clusters in the plasma will increase the output of low- and high-order harmonics. Stable conditions of the QD-containing plasma will allow an investigation of the influence of various properties, in particular plasmon and exciton resonances, on the efficiency of HHG. 

Additionally, THG can be used to study the purely electronic hyperpolarizability of materials. No other mechanism but the non-resonant electron cloud distortion can respond rapidly enough to produce a nonlinear polarization oscillating at the third harmonic. While the effect has been investigated in liquids and solids, the use of thin films comprising QDs and NPs has proved particularly interesting. In spite of the relatively low number of active media in ultrathin films, THG efficiency can become relatively high (of the order of 0.01%). 

Studies of the nonlinear refraction and absorption in other mercury-contained QDs (HgTe, HgSe) have shown that those species demonstrate smaller nonlinear refractive indices and nonlinear absorption coefficients compared with the HgS QDs [20,29]. Moreover, HgS QDs have demonstrated the efficient THG, which was not reported during the studies of HgTe and HgSe QDs.

## 6. Conclusions

In conclusion, the authors synthesized the colloidal mercury sulfide quantum dots and analyzed their low-order NLO properties using femtosecond and nanosecond pulses. HgS QD colloidal suspension showed the strong nonlinear absorption and negative nonlinear refraction while using the 532 nm, 10 ns probe pulses. The NLO parameters of this suspension at 1064 nm were smaller compared with the case of 532 nm probe pulses. The negative nonlinear refraction of HgS QDs was attributed to the Kerr effect in the case of 532 nm and 1064 nm, 10 ns probe pulses, as well as femtosecond pulses.

The thin film containing HgS QDs was studied using the tunable femtosecond laser in the range of 500 to1500 nm. We have shown that these QD-contained films possess strong saturable absorption and negative nonlinear refraction in the case of 400 nm, 150 fs probe pulses. The spectral and intensity variations of saturable absorption and nonlinear refraction of the studied thin films were discussed. The measurements of saturable absorption have shown that these films containing HgS QDs possess the low values of saturated intensity, which is useful for the formation of the low-power femtosecond laser sources. Finally, the authors compared the NLO parameters of these films measured by femtosecond and nanosecond pulses.

The authors demonstrated a high conversion efficiency towards the third harmonic of the 900–1700 nm, 150 fs laser in the thin (70 nm) film containing QDs deposited on the glass substrates. The ratio of TH conversion efficiencies in the films and glasses of the same thickness was estimated to be >10^6^. The intensity, polarization, and spectral dependencies of this process in HgS QD thin film have been analyzed. The third harmonic conversion efficiency was measured to be 7 × 10^−4^. The authors have discussed the relation between the TH process and the low-order NLO properties of these quantum dots, as well as the potential applications of these QDs for HHG.

## Data Availability

The data that support the findings of this study are available on request from the corresponding author.
